# A reanalysis and integration of transcriptomics and proteomics datasets unveil novel drug targets for Mekong schistosomiasis

**DOI:** 10.1038/s41598-024-63869-0

**Published:** 2024-06-05

**Authors:** Charin Thawornkuno, Krittika Srisuksai, Nattapon Simanon, Poom Adisakwattana, Sumate Ampawong, Usa Boonyuen, Yanin Limpanont, Phiraphol Chusongsang, Yupa Chusongsang, Nuttapohn Kiangkoo, Onrapak Reamtong

**Affiliations:** 1 Department of Molecular Tropical Medicine and Genetics, Faculty of Tropical Medicine, Mahidol University, Bangkok, Thailand; 2 Department of Helminthology, Faculty of Tropical Medicine, Mahidol University, Bangkok, Thailand; 3 Department of Tropical Pathology, Faculty of Tropical Medicine, Mahidol University, Bangkok, Thailand; 4 Department of Social and Environmental Medicine, Faculty of Tropical Medicine, Mahidol University, Bangkok, Thailand

**Keywords:** Focal adhesion kinase, Anthelmintic, Schistosomiasis, Drug discovery, Molecular biology

## Abstract

Schistosomiasis, caused by *Schistosoma* trematodes, is a significant global health concern, particularly affecting millions in Africa and Southeast Asia. Despite efforts to combat it, the rise of praziquantel (PZQ) resistance underscores the need for new treatment options. Protein kinases (PKs) are vital in cellular signaling and offer potential as drug targets. This study focused on focal adhesion kinase (FAK) as a candidate for anti-schistosomal therapy. Transcriptomic and proteomic analyses of adult *S. mekongi* worms identified FAK as a promising target due to its upregulation and essential role in cellular processes. Molecular docking simulations assessed the binding energy of FAK inhibitors to *Schistosoma* FAK versus human FAK. FAK inhibitor 14 and PF-03814735 exhibited strong binding to *Schistosoma* FAK with minimal binding for human FAK. In vitro assays confirmed significant anti-parasitic activity against *S. mekongi*, *S. mansoni*, and *S. japonicum*, comparable to PZQ, with low toxicity in human cells, indicating potential safety. These findings highlight FAK as a promising target for novel anti-schistosomal therapies. However, further research, including in vivo studies, is necessary to validate efficacy and safety before clinical use. This study offers a hopeful strategy to combat schistosomiasis and reduce its global impact.

## Introduction

Schistosomiasis is a parasitic disease caused by infection of blood trematode in the genus of *Schistosoma*. More than 250 million people worldwide are infected with schistosomiasis, and it is especially prevalent in Africa. The estimated annual mortality and risk of infection are 280,000 and 732 million cases, respectively, worldwide^1–3^. Currently, there are six species of schistosomes that infect humans, including *S*. *haematobium*, *S*. *mansoni*, *S*. *japonicum*, *S*. *mekongi*, *S*. *guineensis*, *S*. *intercalatum*^4^. Among these, *S. mekongi* is highly prevalent along the Mekong River in southern Lao People’s Democratic Republic (Laos) and northern Cambodia causing Mekong schistosomiasis^5–7^. The majority of the people who acquires *S. mekongi* are villagers who reside near endemic regions. The incidents may occur among immigrants who migrate to other countries. Additionally, *S*. *mekongi* has also been identified among international travelers who have visited areas where it is endemic, making this condition an important travel medicine issue in Southeast Asia^8–10^. Previously, epidemiological studies have shown that the population at risk in the heavily affected endemic areas of Khong Island in Laos, and Kratie province in Cambodia was over 150,000^7^. According to the mass drug administration using praziquantel (PZQ) conducted by the Ministries of Health in Laos and Cambodia the prevalence has gradually decreased to approximately 1500 per year^11^.

The life cycle of the schistosome begins with the eggs being released into the water via feces. Under the influence of water and light, miracidia emerge from these eggs and navigate to a water snail that matches their species. Once inside the snail, the miracidium transforms into a mother sporocyst, which multiplies asexually. Through the daughter sporocyst stages, a large number of cercariae are produced. These cercariae exit the snail by migrating back through its epidermis, becoming the infectious stage for mammals. Humans become infected after contracting these cercarial larvae while swimming in freshwater lakes and rivers^12^. *S*. *mekongi* infection is linked to persistent inflammation in the host’s tissues, resulting in intestinal disease, liver fibrosis, and hepatosplenic inflammation when eggs get trapped in the tissues.

Until now, there is no schistosomiasis vaccine available. Prompt and accurate diagnosis, followed by early treatment, can significantly mitigate the morbidity and mortality risks associated with this disease. Currently, PZQ remains the sole medication recommended for the prevention and treatment of *S*. *mekongi*^13,14^. It has been extensively utilized to manage blood fluke infections and other parasitic diseases in both humans and animals, displaying exceptional effectiveness against numerous worm species^13–15^. However, drug resistance and reduced susceptibility to schistosomiasis treatment have been observed^16–19^. Hence, developing a new class of anti-schistosomal drugs is needed. Numerous researchers are studying conventional drugs that are used to treat various diseases. For instance, administering 150 mg/kg mefloquine, an antimalarial drug, can significantly reduce *S*. *mansoni* eggs in infected mice^20^; however, it has dose-dependent negative effects in children and adults^21^. Artemisinin, the most potent antimalarial drug, and its derivatives such as artemether and artesunate, have been found to have anti-schistosomal activity in several studies conducted on both humans and animals^22–24^. Therefore, it is crucial to develop new and affordable anti-schistosomiasis drugs that differ structurally and functionally from PZQ to combat its rapid development of resistance.

Protein kinases (PKs) are enzymes that transfer a phosphate group to an amino acid in a protein substrate. This process leads to the phosphorylation of the substrate, which plays a crucial role in various cellular processes such as cell proliferation, differentiation, migration, metabolism, and programmed cell death. By catalyzing protein phosphorylation, PKs transmit signals both within and outside the cell, and transfer phosphate groups from ATP to proteins^25,26^. PKs have been identified as important drug targets for several carcinomas and parasitic diseases^27,28^. Recent studies have shown that PKs play a key role in the growth, development, maturation, and survival of *S*. *mansoni*^29–31^, *S*. *japonicum*^32^, indicating their potential as drug targets against *S*. *mekongi*.

In this investigation, PKs were selected from our generated transcriptomic and proteomic datasets, specifically focusing on kinases. The analysis incorporated datasets involving individual adult male and female worms of *S. mekongi*. Candidates for drug targets were identified by analyzing up-regulated PKs from both transcriptomic and proteomic analyses. Subsequent results revealed focal adhesion kinase (FAK), a tyrosine kinase, as a candidate drug target in *S. mekongi*. In silico analysis was employed to identify known compounds targeting FAK, and the chosen compound was subsequently subjected to in vitro testing against *S. mekongi*.

## Results

### Investigation of kinases through transcriptomic and proteomic datasets of *S. mekongi* adult male and female worms

**Figure 1:**
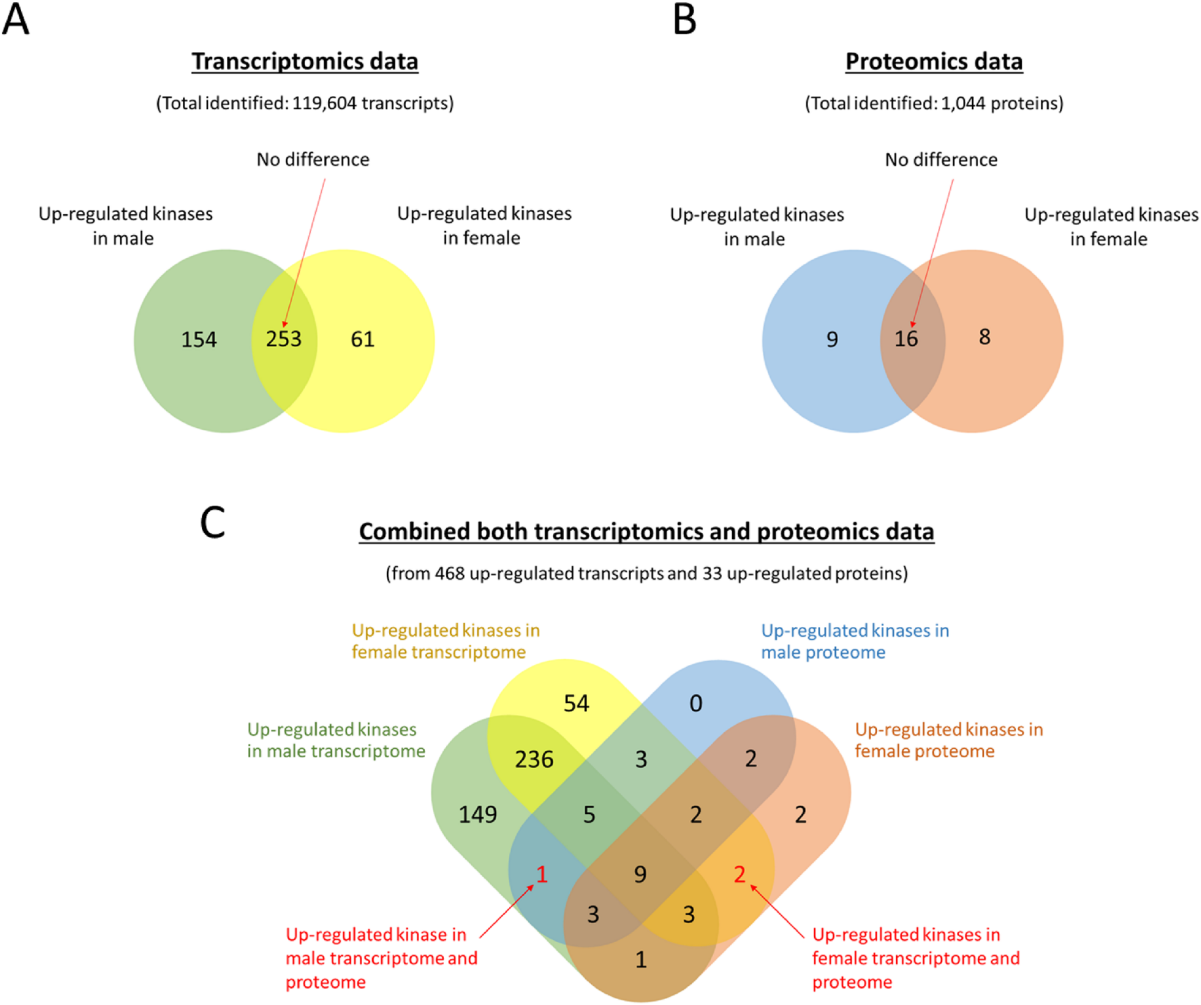
Venn diagram of up-regulated kinases found in transcriptome and proteome of *S. mekongi* male and female worms. (**A**) Venn diagram showing the up-regulated kinases transcripts in both sexes worms. (**B**) Venn diagram showing the most highly abundant kinase proteins in both sexes worms. (**C**) Venn diagram showing the up-regulated and the most highly abundant kinase transcripts and proteins in both sexes of worms.

From our transcriptomics data^63^, we identified a total of 119,604 transcripts in adult male and female *S. mekongi* worms. Among these, 21,833 contigs were associated with at least one of three GO terms in the BLAST database, and 8462 contigs were associated with GO terms in the Pfam database. A total of 407 and 314 kinase transcripts were identified from both male and female worms, respectively (Fig. 1A). In males, pyruvate kinase (EC 2.7.1.40), calmodulin 3b (phosphorylase kinase, delta), and serine/threonine-protein kinase PAK 3 were among the most abundant kinase transcripts. Conversely, in females, pyruvate kinase (EC 2.7.1.40), alpha subunit of casein kinase II (EC 2.7.11.1), and phosphoglycerate kinase (EC 2.7.2.3) being the most abundant kinase transcripts. After comparing kinase expression levels between males and females, 154 kinase transcripts were found to be upregulated in males, including serine/threonine kinase, receptor protein-tyrosine kinase (EC 2.7.10.1), and myosin light chain kinase, smooth muscle. In females, 61 kinase transcripts were upregulated, including connector enhancer of kinase suppressor of ras 2, dual specificity protein kinase CLK2 (EC 2.7.12.1), and protein kinase (Supplementary Dataset 1).

In our proteomics data^64^, we detected a total of 1044 proteins in adult *S. mekongi* adult worms using LC–MS/MS, with 33 of these proteins identified as kinases. In male worms, we identified 25 kinases, with nucleoside diphosphate kinase (EC 2.7.4.6), pyruvate kinase (EC 2.7.1.40), and calmodulin 3b (phosphorylase kinase, delta) being the most abundant kinase proteins. This finding correlates with the transcriptome data. Similarly, in female worms, 24 kinases were identified, with phosphoglycerate kinase (EC 2.7.2.3), alpha subunit of casein kinase II (EC 2.7.11.1), and phosphatidylinositol 4-phosphate 5-kinase type-1 alpha being the most abundant kinase proteins. Notably, the most abundant kinase transcripts and proteins were found to be correlated. Based on comparison of kinase expression between male and female, nine kinases were the most highly abundant proteins in males, including nucleoside diphosphate kinase (EC 2.7.4.6), pyruvate kinase (EC 2.7.1.40), and LOK-like protein kinase. In female worms, eight kinases were the most highly abundant proteins, including phosphatidylinositol 4-phosphate 5-kinase type-1 alpha, phosphoglycerate kinase (EC 2.7.2.3), and alpha subunit of casein kinase II (EC 2.7.11.1) (see Fig. 1B and Supplementary Dataset 2 for more details).

This study integrated transcriptomics and proteomics data to analyze the differentially expressed kinase transcripts and proteins in adult *S*. *mekongi* worms (refer to Fig. 1C). Ultimately, only one kinase in male worms (tyrosine kinase; UniProt entry: G4VP68) and two kinases in female worms (alpha subunit of casein kinase II (EC 2.7.11.1); UniProt entry C1L7H2 and phosphoglycerate kinase (EC 2.7.2.3); UniProt entry C1LT16) showed consistent up-regulation in both transcriptomics and proteomics results. The pairing of male and female worms is essential for the development and reproduction of female worms, leading to egg production and fecundity, which contributes to pathogenesis. Inhibiting *Schistosoma* maturation and egg production could reduce pathogenesis and transmission to the host. Therefore, these three kinases are considered candidate targets for developing a new drug against adult *S*. *mekongi* worms.

### Drug target prediction and prioritization

**Figure 2:**
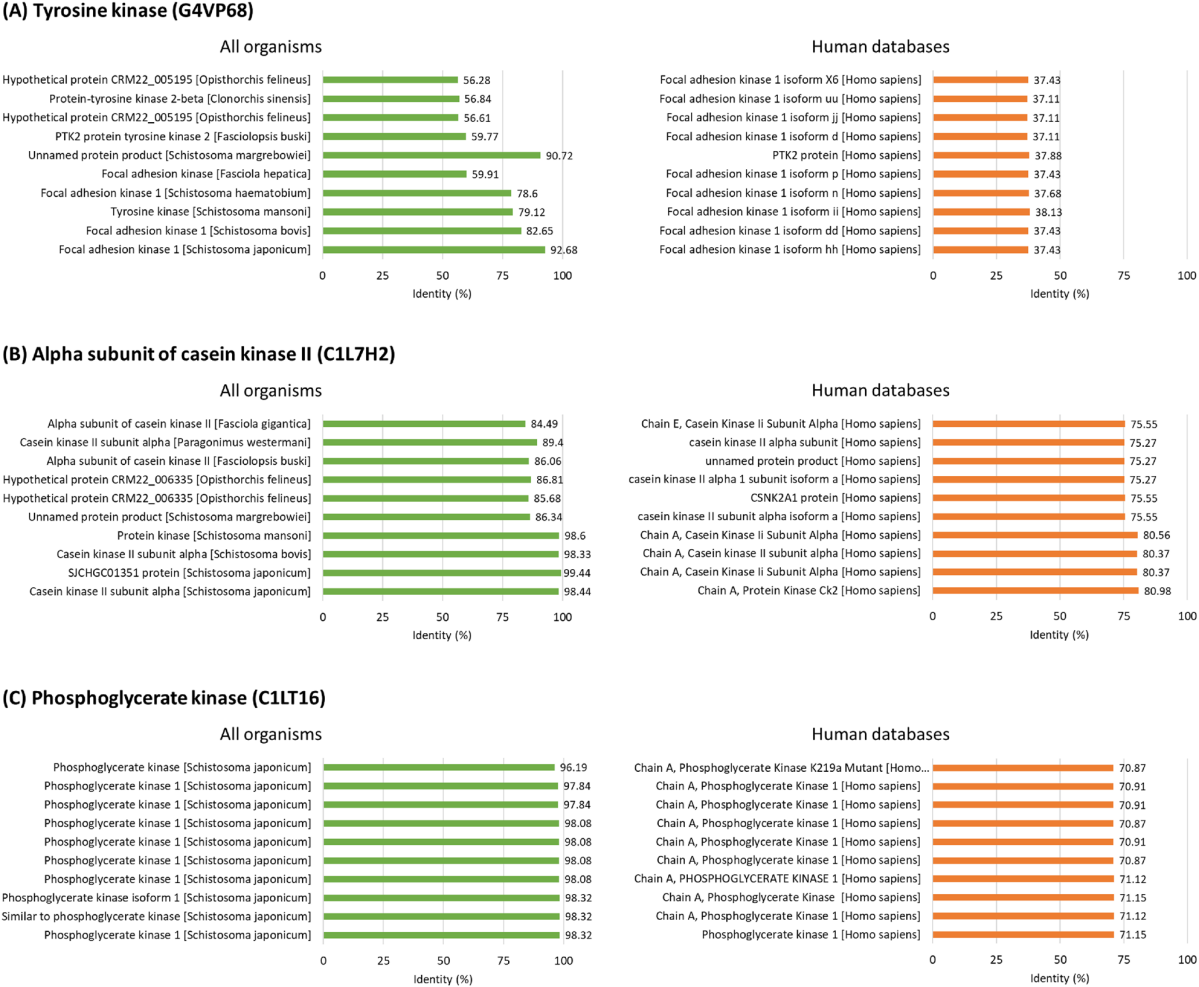
Drug target prediction and prioritization. (**A**) Pairwise alignments of tyrosine kinase (G4VP68) against all organisms and human using the BLASTP algorithm. (**B**) Pairwise alignments of the alpha subunit of casein kinase II (C1L7H2) against all organisms and human using the BLASTP algorithm. (**C**) Pairwise alignments of phosphoglycerate kinase (C1LT16) against all organisms and human using the BLASTP algorithm.

Pairwise alignments of three candidate kinases were analyzed to predict and prioritize a drug target. The full protein sequences were retrieved from the transcriptomics database and were searched against all organisms and humans using the BLASTP algorithm. The top-10 sequence similarity (% identity) of each kinase was shown in Supplemental Dataset 3. The similarity following search against all organisms in the database was 56.28–92.72%, 84.49–98.44%, and 96.19–98.32% for a focal adhesion kinase (FAK), the alpha subunit of casein kinase II, and phosphoglycerate kinase, respectively. All the proteins identified from the similarity search against all organisms belonged to helminthic proteins, including *Opisthorchis felineus*, *Clonorchis sinensis*, *Fasciolopsis buski*, *Schistosoma* spp, *Fasciola* spp., and *Paragonimus westernmani*. Furthermore, the similarity following the search against human proteins in the database, 75.27–80.98%, and 70.87–71.15% identity, was identified from the alpha subunit of casein kinase II and phosphoglycerate kinase, respectively. Only 37.11–38.13% identity was identified from tyrosine kinase following a search against the human proteins database (Fig. 2). According to the high similarity to worm proteins and low similarity to the human protein, FAK is considered a drug target for drug development.

### Binding energy analysis

As mentioned earlier, FAK is a good candidate for developing an anti-*S*. *mekongi* drug. It is important to ensure that the compound chosen has a lower binding energy for FAK in helminths rather than in humans. Therefore, molecular modeling and docking were used to determine the binding energy of the candidate chemical compounds against FAK protein from both humans and other organisms. A more negative binding energy indicates a stronger binding of the chemical compound to the targeted protein. The higher Δ(Human − *x̄*_*Schis*_) indicated the binding of the selected compound to human FAK was weaker than that of FAK from *Schistosoma* species, suggesting that the chemical compound's selectivity is more towards *Schistosoma* than human.

**Figure 3:**
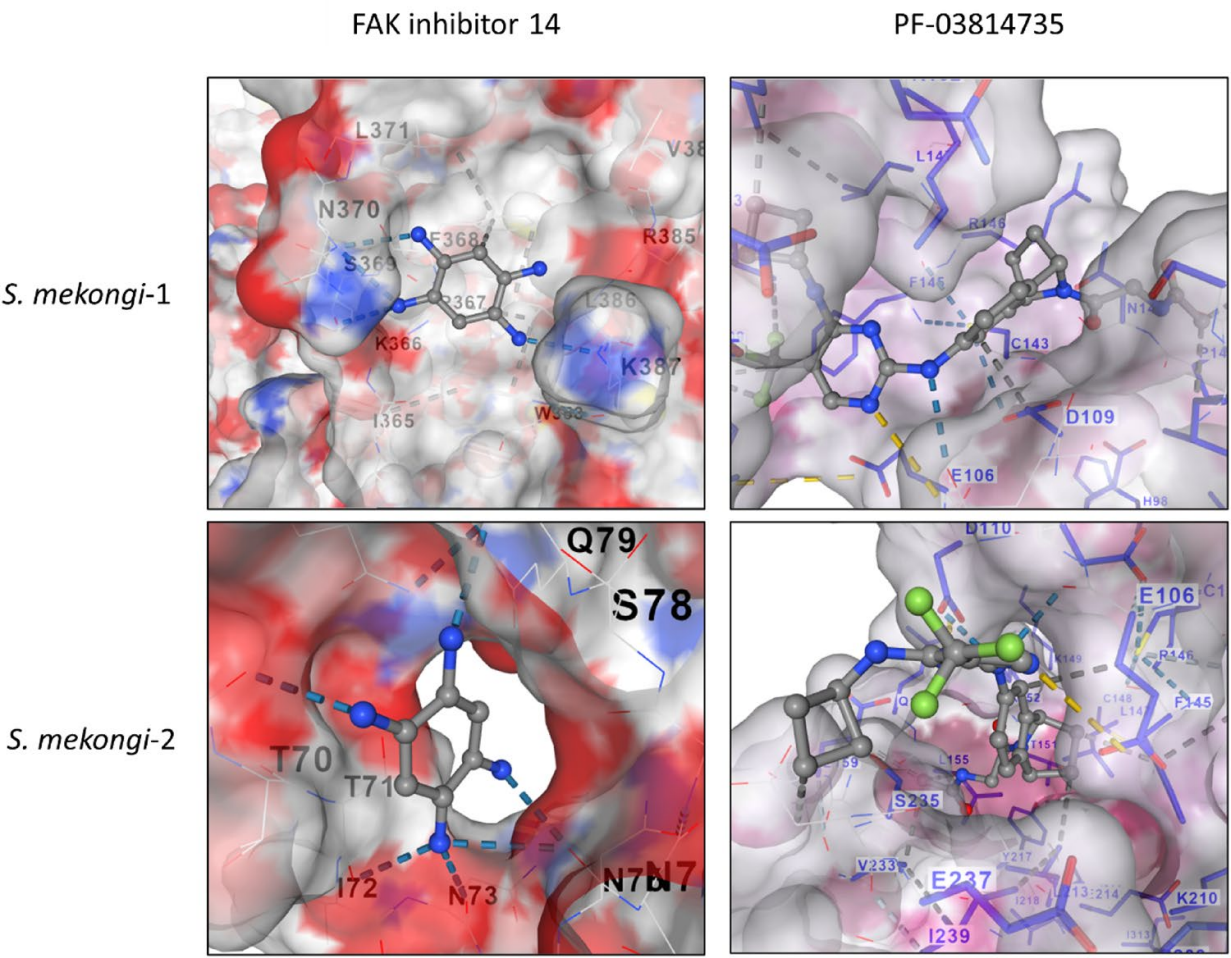
Docking of FAK inhibitor 14 and PF-03814735 to the tyrosine kinase of human and *Schistosoma* species. The left panel depicts FAK inhibitor 14 binding, while the right panel depicts PF-03814735 binding.

The molecular docking was conducted between 10 known FAK inhibitors, including PF-562271, FAK inhibitor 14, PF-573228, PF-431396, Y11, PND-1186, TAE-226, PF-03814735, BI-4464 and GSK2256098 against FAK proteins from *S*. *mansoni* (A0A3Q0KMP3), *S*. *japonicum* (A0A4Z2CUR2), human (Q59GN8), and two FAK proteins from *S*. *mekongi* (derived from transcriptomics data). The outcomes are presented in Table 1. As per the docking results, PF-03814735 (0.57 kcal/mol) and FAK inhibitor 14 (0.26 kcal/mol) demonstrated weaker binding to human proteins and a preference for *Schistosoma* FAK. The differential free energy of binding between these compounds and the average of the four *Schistosoma* FAK proteins was notably high, indicating their potential as strong candidates. The docking interactions of FAK inhibitor 14 and PF-03814735 with *S. mekongi*_1 and *S. mekongi*_2 are illustrated in Fig. 3. These two compounds were subsequently employed for anthelminthic activity. From these results, it has been suggested that FAK inhibitor 14 and PF-03814735 exhibited the most promising potential for further in vitro assessment.

**Table 1:** Binding energy of the candidate chemical compounds against focal adhesion kinase (FAK) protein from human and *Schistosoma* species.

No	Compound name	PubChem CID number	Chemical structure	Binding energy (kcal/mol)						
				Humans	*S*. *mansoni*	*S*. *japonicum*	*S*. *mekongi*-1	*S*. *mekongi*-2	*x̄*_*Schis*_	Δ(Human − *x̄*_*Schis*_)
1	PF-03814735	51346455	C_23_H_25_F_3_N_6_O_2_	− 7.99	− 8.42	− 8.71	− 9.11	− 7.99	− 8.56	0.57
2	FAK inhibitor 14	78260	C_6_H_14_C_l4_N_4_	− 4.16	− 4.23	− 4.91	− 4.18	− 4.36	− 4.42	0.26
3	GSK2256098	46214930	C_20_H_23_ClN_6_O_2_	− 7.67	− 8.01	− 9.1	− 7.15	− 7.19	− 7.86	0.19
4	BI-4464	58522530	C_28_H_28_F_3_N_5_O_4_	− 8.19	− 8.18	− 8.24	− 8.04	− 8.74	− 8.3	0.11
5	PF-573228	11612883	C_22_H_20_F_3_N_5_O_3_S	− 8.1	− 8	− 9.2	− 7.94	− 7.57	− 8.18	0.08
6	TAE-226	9934347	C_23_H_25_ClN_6_O_3_	− 8.19	− 8.47	− 8.69	− 8.07	− 7.84	− 8.27	0.08
7	PND-1186	25073775	C_25_H_26_F_3_N_5_O_3_	− 8.98	− 8.56	− 8.7	− 8.31	− 7.32	− 8.22	− 0.76
8	PF-431396	11598628	C_22_H_21_F_3_N_6_O_3_S	− 9.78	− 7.41	− 8.28	− 8.88	− 7.29	− 7.97	− 1.82
9	PF-562271	16118986	C_27_H_26_F_3_N_7_O_6_S_2_	− 9.45	− 6.81	− 7.97	− 7.67	− 7.3	− 7.44	− 2.01
10	Y11	24195918	C_8_H_17_BrN_4_O	− 10.66	− 6.71	− 5.49	− 6.08	− 6.01	− 6.07	− 4.59

*x̄*_*Schis*_ indicated mean binding energy among *Schistosoma* species. Δ(Human − *x̄*_*Schis*_) indicated the different binding energy between humans and the mean binding energy of all *Schistosoma* species.

### In vitro evaluation of selected compounds

**Figure 4:**
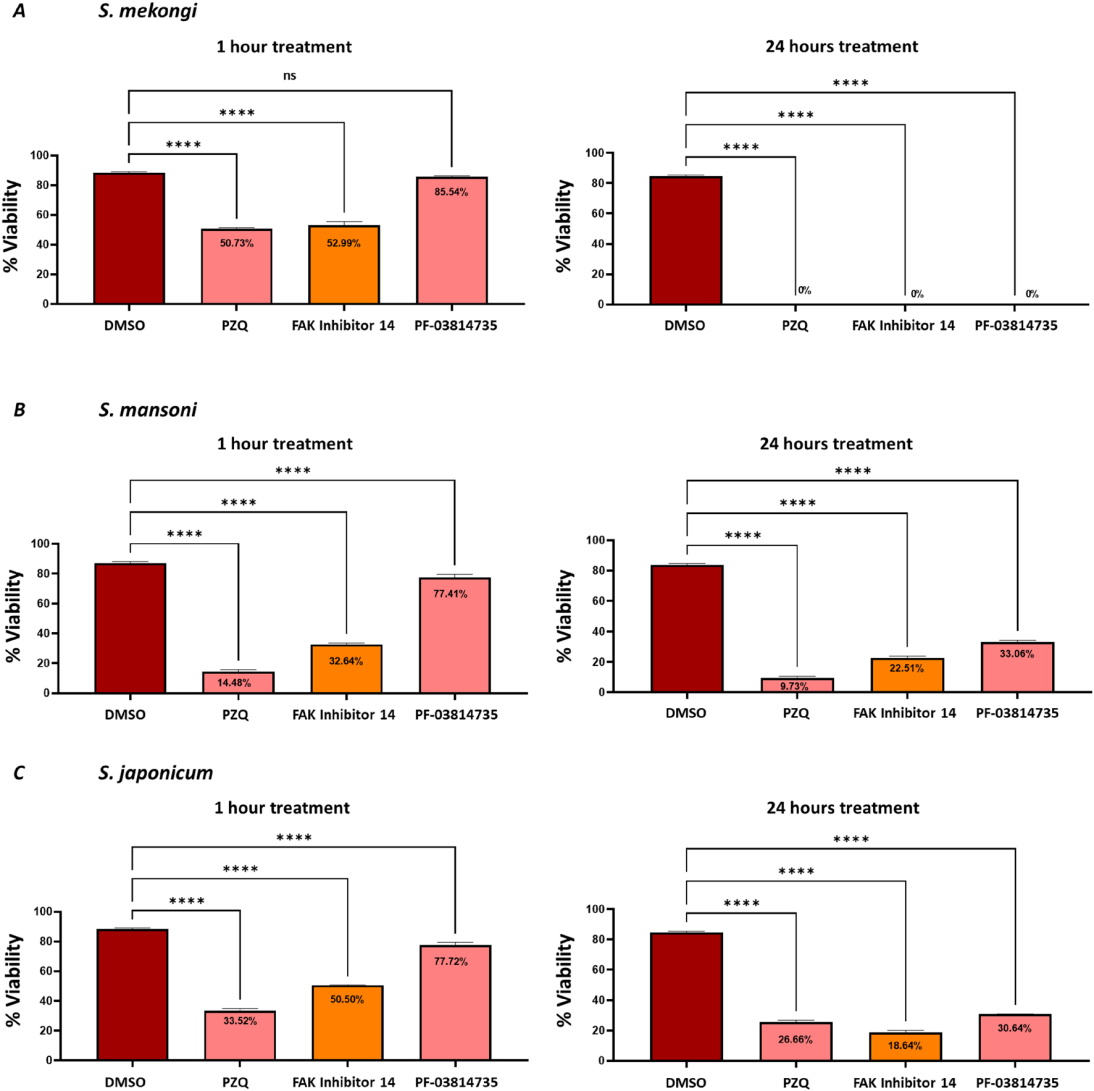
In vitro evaluation of selected compounds. Worm viability was monitored under an inverted microscope at 1 h and 24 h after being treated with PZQ (128 µM), FAK inhibitor 14 (140 µM), and PF-03814735 (84 µM). Data were shown as the mean ± S.E.M. two-way ANOVA was used (* significance level at P < 0.05). The results were done in triplicates.

In vitro testing assessed the anti-schistosomal activity of FAK inhibitor 14 against *S. mekongi*, *S*. *mansoni*, and *S*. *japonicum*. Based on our previous study, *S. mekongi* showed that the half maximal inhibitory concentration (IC_50_) of PZQ was 4 µg/mL after one hour of treatment^33^. Consequently, this study utilized the same concentration of 40 µg/mL to assess the anthelmintic activity of FAK inhibitors. Results indicate that, within an hour of treatment, the viability of *S. mekongi*, *S. mansoni*, and *S. japonicum* significantly decreased to 52.99%, 32.64%, and 50.50%, respectively. After 24 h, the maximum activity was observed with viability of 0%, 22.51%, and 18.64% in *S*. *mekongi*, *S*. *mansoni*, and *S*. *japonicum*, respectively (Fig. 4). PF-03814735 also showed a significant decrease in viability with *S*. *mansoni* and *S. japonicum*, dropping to 77.41% and 77.72% after 1 h. After 24 h, the maximum activity was observed with viability of 0%, 33.06%, and 30.64% in *S*. *mekongi*, *S*. *mansoni*, and *S*. *japonicum*, respectively (Fig. 4). These results were consistent with those of PZQ, which showed a significant decrease in viability within an hour of treatment, with *S*. *mekongi*, *S*. *mansoni*, and *S*. *japonicum* decreasing to 50.73%, 14.48%, and 33.52%, respectively. After 24 h, the maximum activity was observed with viability of 0%, 9.73%, and 26.66% in *S*. *mekongi*, S. *mansoni*, and *S*. *japonicum*, respectively (Fig. 4).

Cytotoxicity tests were conducted on two substances, FAK inhibitor 14 and PF-03814735, and compared to PZQ in the human liver cancer cell line (HepG2). The results showed that the 50% cytotoxic concentration (CC_50_) of FAK inhibitor 14 and PF-03814735 was 11.2 ± 1.1 and 7.6 ± 0.4 µM on the HepG2 cells line, respectively. In addition, PZQ demonstrated lower cytotoxicity at CC_50_ of 157.3 ± 5.1 µM on the HepG2 cell line (Table 2).

**Table 2:** Cytotoxicity testing of the selected compounds compared to PZQ.

Compound	CC_50_ (µM)
PZQ	157.3 ± 5.1
FAK inhibitor 14	11.2 ± 1.1
PF-03814735	7.6 ± 0.4

CC_50_ indicates the 50% cytotoxic concentration of the chemical compound against the HepG2 cell line. Results are reported as mean ± S.E

### Immunohistochemistry

**Figure 5:**
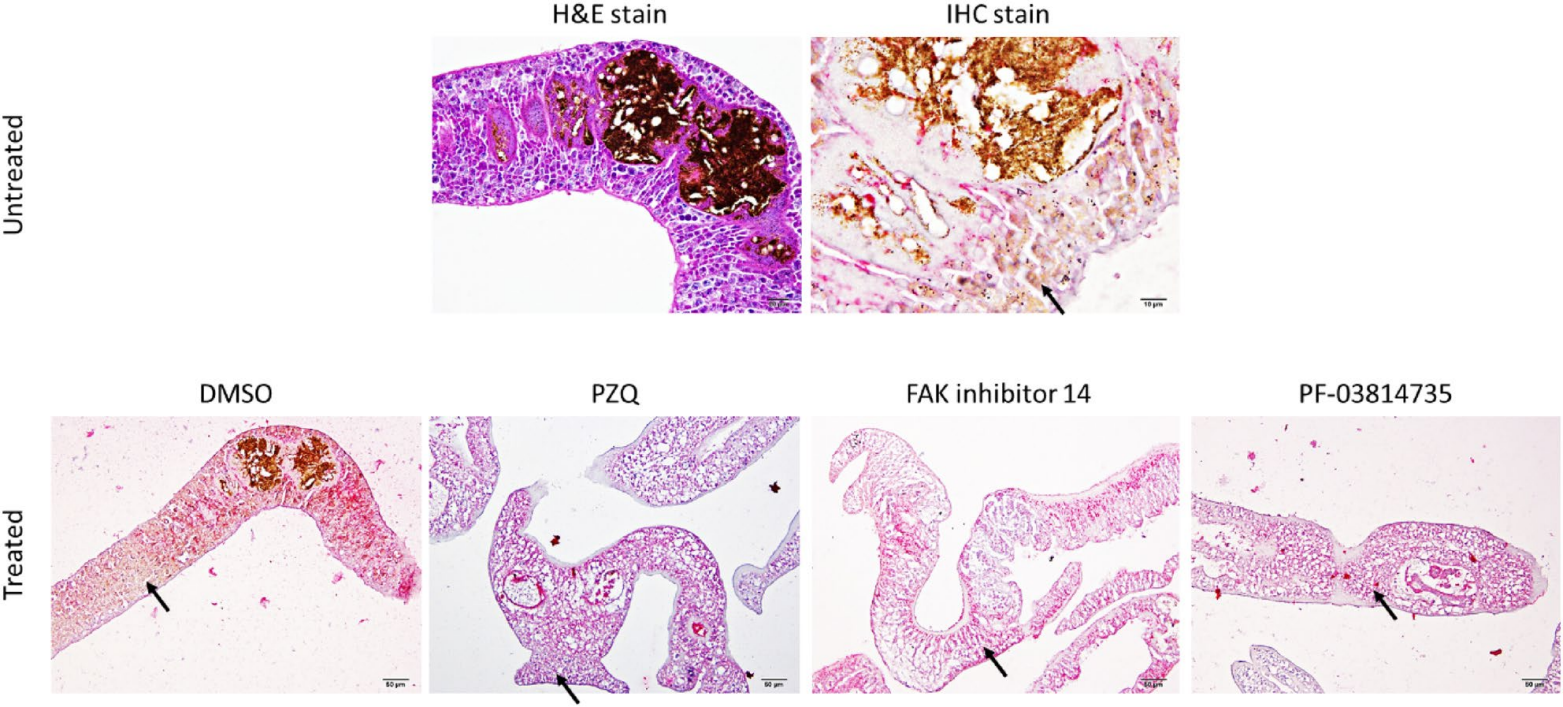
Histological (H&E) and immunohistochemical (IHC) staining of male *S. mekongi* treated with different chemicals. Upper panel: H&E and IHC staining of untreated parasites illustrated internal structures, including the integument, parenchymal cells, and gut. Immunolocalization of phosphoserine in the parasites indicates the positive immunolabelling of liquid permanent red, mainly in the parenchymal cells and integument throughout the worm. Lower panel: comparative study of phosphoserine expression in the parasites treated with or without FAK inhibitors: DMSO, FAK inhibitor 14, PF-03814735, and PZQ. Phosphorylation is indicated by black arrows.

FAK is a nonreceptor protein kinase playing an important role in a variety of cellular processes including growth, motility, differentiation, and metabolism. According to the phosphorylation sites in eukaryotic organisms occur mainly on serine, threonine, and tyrosine residues at a ratio of 1800:200:1^34^. Moreover, FAK undergoes phosphorylation on both tyrosine and serine residues. To elucidate the distribution of phosphoproteins following treatment with FAK inhibitors, anti-phosphoserine antibodies were employed to visualize phosphoproteins in male *S*. *mekongi* adult worm tissue. The results showed that integument, parenchymal cells and gut of male worms were highly phosphorylated in untreated and dimethyl sulfoxide (DMSO) treated male worms. In contrast to treated worms, lower phosphorylation was observed after being treated with PZQ, FAK inhibitor 14 and PF-03714735 (Fig. 5).

## Discussion

This study employed transcriptomic and proteomic datasets to investigate kinase expression in *S*. *mekongi*, aiming to deepen our understanding of molecular biology and identify potential drug targets. In males, transcriptomics identified pyruvate kinase, calmodulin 3b, and serine/threonine-protein kinase PAK 3 as the most abundant kinase transcripts. Whereas proteomics identified nucleoside diphosphate kinase, pyruvate kinase, and calmodulin 3b as the most abundant kinases in males. Pyruvate kinase was the most abundant kinase transcript and protein in both of male and female worms. Pyruvate kinase is an enzyme that facilitates the conversion of phosphoenolpyruvate and ADP into pyruvate and ATP during glycolysis, playing a key role in cellular metabolism regulation^35^. In parasite, pyruvate kinase is a crucial enzyme in energy production and is considered a potential drug target. *T*. *spiralis* pyruvate kinase M (TsPKM) plays role in sugar metabolism, larval molting, and development. TsPKM, identified with essential functional domains and a tetrameric structure, showed specific localization in various tissues and developmental stages of *T*. *spiralis*. Silencing TsPKM gene expression significantly impacted enzyme activity, larval molting, sugar metabolism, and overall parasite growth, suggesting TsPKM as a promising therapeutic target for trichinellosis treatment^36^. Due to its abundance in both genders, pyruvate kinase in *S*. *mekongi* emerges as a promising candidate for drug development, similar to its role observed in *T*. *spiralis*. Calmodulin 3b exhibited high expression levels in male *S*. *mekongi*. Calmodulin is a vital intracellular protein that binds calcium ions (Ca^2+^) and acts as a key second messenger, facilitating various biological activities through extensive interactions with proteins and peptides^37^. Calmodulin controls the contractile activity of smooth muscle. Its primary function in smooth muscle involves activating crossbridge cycling and promoting force development in response to transient increases in intracellular calcium concentration achieved through the activation of myosin light-chain kinase and subsequent phosphorylation of myosin^38^. The high expression of calmodulin, a protein involved in movement, correlates with the schistosome male worm's specialized role of enveloping the female in a gynecophoral canal and transporting her through the venous system while she deposits eggs^39^. Serine/threonine-protein kinase PAK 3 was identified as a highly expressed transcript in male *S. mekongi*. In *S. mansoni*, serine/threonine-protein kinase has been identified as a potential drug target^40^; however, the functional understanding of this protein in schistosomes remains limited. Nucleoside diphosphate kinase was identified as the abundant protein in male *S. mekongi*. Nucleoside diphosphate kinases are oligomeric proteins responsible for synthesizing nucleoside triphosphates^41^. Several studies have described the interaction between nucleoside diphosphate kinase and cytoskeletal components, including actin, actin-binding proteins, intermediate filaments, and tubulin. This interaction is considered highly relevant in the context of metastasis, attributed to the well-established role of the cytoskeleton in cell motility and the spread of metastasis^42,43^. The elevated expression of nucleoside diphosphate kinase in adult male *S*. *mekongi* may be associated with the specialized function of transporting the female schistosome through the host venous system.

In females, transcriptomics revealed pyruvate kinase, alpha subunit of casein kinase II, and phosphoglycerate kinase as the most abundant kinase transcripts. Similarly, proteomics identified phosphoglycerate kinase, alpha subunit of casein kinase II, and phosphatidylinositol 4-phosphate 5-kinase type-1 alpha as the most abundant kinases in females. The alpha subunit of casein kinase II was identified as the abundant kinase in female *S*. *mekongi* based on both transcriptome and proteome datasets. Cytosols from *C*. *elegans* embryos and gravid adults, which include fertilized eggs and embryos, show enrichment in casein kinase II activity. In contrast, cytosols from newly hatched larvae, subsequent larval stages, and immature adults display casein kinase II levels that are 3-ten fold lower compared to embryo cytosols^44^. This protein plays a role in the reproduction of *C*. *elegans*. Similarly, the high expression of the alpha subunit of casein kinase II in *S. mekongi* may be involved in the fertilization and embryogenesis process of the female adult worm. Phosphatidylinositol 4-phosphate 5-kinase type-1 alpha was identified as an abundant kinase in females based on proteomics data. However, there is limited information available about this kinase in parasites.

In *S*. *mekongi*, differential expression of kinases was observed in both transcriptome and proteome data. In the transcriptome, serine/threonine kinase, receptor protein-tyrosine kinase (EC 2.7.10.1), and myosin light chain kinase, smooth muscle were among the most up-regulated in males. Conversely, in the proteome data, connector enhancer of kinase suppressor of ras 2, dual specificity protein kinase CLK2 (EC 2.7.12.1), and protein kinase were identified as the most highly abundant kinases in males. Serine/threonine kinases are enzymes responsible for transferring a phosphate group from ATP to a protein substrate, specifically targeting serine or threonine amino acid residues. These kinases play crucial roles in regulating cell proliferation, differentiation, and secondary metabolism^45^. The activity of serine-threonine kinases is linked to the *S*. *mansoni* surface, where surface proteins undergo phosphorylation on serine and threonine residues under in vitro conditions. No notable tyrosine phosphorylation of surface molecules was observed^46^. Based on this evidence, parasites are proposed to respond to external stimuli from the host and from schistosomes of the opposite sex via serine-threonine kinases. The increased expression of serine-threonine kinases in *S*. *mekongi* male is associated with the female schistosome residing within the ventral groove, or gynaecophoric canal, of the male. The male, being more exposed to the host environment, engages in greater interactions with the host. Furthermore, the stimulus provided by the male worm is essential not only for the female’s physical and reproductive development but also for maintaining her mature state^47^. This ongoing interaction by the male schistosome is crucial for the female’s well-being and development. The receptor protein-tyrosine kinase and myosin light chain kinase specific to smooth muscle were found to be significantly abundant in male *S*. *mekongi*. Receptor tyrosine kinases are vital elements of signal transduction pathways facilitating cell-to-cell communication. These single-pass transmembrane receptors bind polypeptide ligands, primarily growth factors, and are pivotal in regulating cellular growth, differentiation, metabolism, and motility^48^. Smooth muscle plays a critical role in maintaining homeostasis across various body functions and responds adaptively to stresses caused by pathological conditions. Through identified cell signaling networks, numerous potential mechanisms have been identified for initiating smooth muscle contraction, both with and without myosin regulatory light chain phosphorylation mediated by myosin light chain kinase^49^. Thus, the presence and activity of receptor protein-tyrosine kinase and myosin light chain kinase are crucial for mobility and muscle contraction of *S. mekongi* male. This aligns with the specialized role of the schistosome male worm, which envelops the female within a gynecophoral canal and transports her through the venous system. From proteomics data, it was observed that the connector enhancer of kinase suppressor of ras 2 and dual specificity protein kinase CLK2 were also abundant in males. However, the functions of these kinases are not well understood. In females, according to transcriptome data, nucleoside diphosphate kinase (EC 2.7.4.6), pyruvate kinase (EC 2.7.1.40), and LOK-like protein kinase were the most highly abundant. Additionally, in the proteome data, phosphatidylinositol 4-phosphate 5-kinase type-1 alpha, phosphoglycerate kinase (EC 2.7.2.3), and alpha subunit of casein kinase II were identified as the most highly abundant proteins. LOK-like protein kinase as a unique serine/threonine kinase. In mouse, LOK-like protein kinase may participate in a new signaling pathway that is different from the established MAP kinase cascades^50^. However, limited information is available regarding these aspects in parasites. Phosphoglycerate kinase is a glycolytic enzyme^51^. The initial production of ATP in glycolysis is mediated by the enzyme phosphoglycerate kinase. In *S*. *japonicum*, phosphoglycerate kinase could be a crucial and indispensable protein for schistosome survival and development^52^. Vaccination with *Fasciola hepatica* phosphoglycerate kinase provided partial protection against infection, attributed to the disruption of the fluke's energy metabolism^53^. This protein likely plays a critical role in energy metabolism, which is important for egg production and reproduction in female *S. mekongi*.

The pairing of male and female worms is critical for the development and reproduction of female worms, resulting in egg production and fecundity, which contribute to pathogenesis. Inhibiting *Schistosoma* maturation and egg production could decrease pathogenesis and transmission to the host. In transcriptomic data, there were nine transcripts of tyrosine kinase in male including A0A094ZJ79, G4VP68, G4V7A7, G4LX52, G4VLP0, A0A095ANK3, A0A094ZYZ4, G4V7A7, Q7YSY1. While, there were two transcripts of tyrosine kinase in female including C7TXQ4, G4V7A7. In proteomics data, there was one tyrosine kinase in male worm but cannot found in female. The absent of tyrosine kinase in proteome data might caused from their low abundance to be detected. Interstingly, FAK was found to be abundant in males based on transcriptome and proteome data. Due to its high similarity to worm proteins and low similarity to human proteins, FAK is considered a promising drug target for drug development. FAK is a vital signaling molecule activated by various stimuli, acting as a biosensor or integrator to regulate cell motility. FAK exerts its influence through intricate molecular interactions, impacting the cytoskeleton, cell adhesion structures, and membrane protrusions to modulate cell movement^54^. Targeting FAK is regarded as a promising strategy for cancer therapy using small molecules. Several FAK inhibitors have been identified as anticancer agents with diverse mechanisms of action^55^. In *S*. *mansoni*, FAK is classified into cytoplasmic tyrosine kinase. Tyrosine kinase explores as potential targets for the development of novel anti-schistosome drugs^56^. Sorafenib, a multi-targeted tyrosine kinase inhibitor, has been shown to significantly suppress *S*. *japonicum*-induced liver fibrosis in mice when combined with PZQ^57^. An intriguing novel computational drug repurposing pipeline has identified a range of tyrosine kinase inhibitors, originally designed as anti-cancer drugs, that could serve as potential new schistosomicidal agents. These include imatinib, bosutinib, crizotinib, nilotinib, and dasatinib^58^. These studies reinforce our discovery that FAK is a critical target for the development of schistosomiasis drugs.

Two compounds, FAK inhibitor 14 and PF-03814735, have exhibited promising potential as treatments for Schistosomiasis. FAK inhibitor 14 (1,2,4,5-benzenetetramine tetrahydrochloride) is a potent and specific inhibitor of focal adhesion kinase. It effectively inhibits the autophosphorylation activity of FAK, reduces the viability of cancer cells, and impedes tumor growth^59^. FAK inhibitor 14 effectively inhibited the growth of breast, pancreatic, and neuroblastoma tumors. Additionally, FAK inhibitor 14 demonstrated antitumor activity in glioblastoma and colon cancer models^60^. PF-03814735 is an innovative, potent, orally bioavailable, reversible inhibitor targeting both Aurora1 and Aurora2 kinases. It is currently undergoing phase I clinical trials for the treatment of advanced solid tumors. Although FAK inhibitor 14 and PF-03814735 had higher cytotoxicity than PZQ, these drugs are currently being assessed in Phase I as explained above. In intact cells, PF-03814735 effectively inhibits the activity of Aurora1 and Aurora2 kinases, leading to decreased levels of phospho-Aurora1, phosphohistone H3, and phospho-Aurora2. This inhibition results in a blockade of cytokinesis, thereby halting cell proliferation and promoting the formation of polyploid multinucleated cells^61^. Drug repositioning involves identifying new applications for existing drugs. This approach is crucial as it leverages the well-understood pharmacokinetics, pharmacodynamics, and toxicity profiles of these drugs, thereby reducing the risk of future failures and cutting down on development costs and approval timelines. Anthelmintics, traditionally used as antiparasitic drugs, have recently demonstrated promising anticancer effects in both laboratory and animal studies^62^. Conversely, leveraging anticancer drugs for their potential anthelmintic properties could be advantageous. FAK inhibitor 14 and PF-03814735 have not yet been evaluated for their anthelminthic activity. In this study, in vitro studies have demonstrated that these compounds significantly reduce the viability of *S. mansoni*, *S*. *japonicum*, and *S*. *mekongi*, comparable to the effectiveness of PZQ, the current standard treatment for schistosomiasis. These findings suggest that FAK inhibitor 14 and PF-03814735 could potentially serve as alternative or adjunctive therapies for schistosomiasis. Nonetheless, further investigations are required to assess their safety and in vivo efficacy before considering them as viable treatment options. Overall, these compounds represent a promising approach for treating Schistosomiasis, potentially offering novel mechanisms of action.

## Conclusion

Schistosomiasis, caused by *Schistosoma* trematodes, is a major global health concern, particularly in Africa and Southeast Asia. The rise of PZQ resistance underscores the need for new treatments. This study targeted PKs, focusing on FAK, identified through transcriptomic and proteomic analyses of *S. mekongi*. Molecular docking revealed two FAK inhibitors, FAK inhibitor 14 and PF-03814735, with strong binding to *Schistosoma* FAK and low binding energy for human FAK. Both compounds significantly reduced parasite viability in vitro with minimal toxicity to human cells, showing promise as safe alternatives to PZQ. Further research, including in vivo studies, is essential to validate their efficacy and safety for clinical use. This study offers a potential avenue for combating schistosomiasis and reducing its global impact.

## Method details

### Identification of kinases from *S. mekongi* transcriptomic and proteomic datasets

Transcriptomic^63^ and proteomics^64^ datasets from both male and female *S*. *mekongi* adult worms were obtained from our recent work. For the transcriptomics dataset, a total of 119,604 transcripts were identified. Among these transcripts, kinases were identified based on their association with at least one GO term in the BLAST or Pfam databases. To analyze the differentially expressed transcripts, transcripts per million (TPM), fragments per kilobase of exon per million fragments mapped (FPKM), credibility intervals and expected counts were computed using RSEM (v.1.3.0)^65^. Transcripts with expression values of more than 1 count per-million (cpm) were extracted for differential expression analysis. Analysis of DE transcripts comparing male vs female worms was conducted using edgeR v.3.20.7. Finally, the expression fold change between sexes were calculated and log-transformed. In the proteomics dataset, a total of 1044 proteins were identified in adult *S*. *mekongi* worms using LC–MS/MS. The proteins classified as kinases were gathered based on identification from the transcriptomics database. The quantification of kinase proteins was calculated in exponentially modified protein abundance index (emPAI)^66^. This comprehensive analysis provides detailed insights into the transcriptomic and proteomic profiles of *S*. *mekongi* worms, particularly focusing on kinase expression patterns and differential regulation between male and female worms.

### Compound selection and in silico analysis

The protein sequences of *S*. *mansoni* (XP_018653840.1), *S*. *japonicum* (TNN08032.1), and human (NP_001339669.1) were obtained from the NCBI database. The *S*. *mekongi* protein sequence was from the transcriptome database. The structural models of FAK of *S*. *mansoni* (A0A3Q0KMP3), *S*. *japonicum* (A0A4Z2CUR2), and human (Q59GN8), were retrieved from AlphaFold protein structure database (https://alphafold.ebi.ac.uk/), while the structural models of FAK of *S*. *mekongi* were built by Swiss-model (https://swissmodel.expasy.org/) using *S*. *japonicum* (A0A4Z2CUR2) as the template^67^. The models were subjected to energy minimization using YASARA minimization^68^ server and evaluated by SAVESv6.0—structure validation server (https://saves.mbi.ucla.edu/)^69^. The PDB-files were converted into PDBQT format (input for AutoDockTools software). The grid search size was selected as 275 × 275 × 275 Å. AutoDockTools software version 1.5.6 was used to perform molecular docking between compounds and proteins^70^. The chemical structures of FAK inhibitors were retrieved from the PubChem database (https://pubchem.ncbi.nlm.nih.gov/). Three-dimensional structures of chemical compounds were generated as PBD-files using SMILES translator web-based software (https://cactus.nci.nih.gov/translate/). Docking parameters were set to default except for the following: 50 runs and 200 for a number of individuals in the population. Lamarckian genetic algorithm was chosen for the free energy of binding calculation. The graphical molecular surface representations of the FAK-ligand complexes were visualized using BIOVIA Discovery Studio Visualizer.

### In vitro anthelmintic testing

All experiments in the animal study were approved by the Faculty of Tropical Medicine-Animal Care and Use Committee (FTM-ACUC) with the certificate number FTM-ACUC 032/2020. All animal experiments were conducted in accordance with ARRIVE guidelines. All methods were performed in accordance with relevant guidelines and regulations. The laboratory strains of *S*. *mekongi*, *S*. *mansoni*, and *S*. *japonicum* used in this study were maintained in the Animal Care Unit of the Faculty of Tropical Medicine at Mahidol University. In summary, *S*. *mekongi*, *S*. *mansoni*, and *S*. *japonicum* cercariae at 4 weeks post-infection. Each ICR mouse (Mus musculus) was then exposed to 25–30 cercariae on the abdomen using a hairpin loop. The infected mice were kept for 8 weeks, after which adult worms were collected via vascular perfusion using 0.85% normal saline solution. These worms were cultured in RPMI 1640 purchased from Hyclone (Logan, UT, USA) for anthelmintic testing. All chemicals, including PZQ, FAK inhibitor 14, and PF-03814735, were initially mixed with DMSO and then diluted with RPMI medium to final concentration at 40 μg/mL. This concentration was IC_50_ of PZQ to *S*. *mekongi*^33^. This mixture was administered for 1 h to 10 pairs of *S. mekongi* at each concentration (with three biological replicates per concentration). The viability of the worms was assessed based on their movement. Using a video microscope (Celestron, CA, USA) and worms that remained motionless for 1 min were considered dead. After the hour-long treatment, all worms were collected 10% neutral buffer formalin for further immunohistochemistry analysis.

### Cytotoxicity testing

HepG2 was used for conducting a cytotoxicity assay on each compound. The HepG2 cells were cultured in a 96-well plate using Eagle’s minimum essential medium (MEM) supplemented with 10% fetal bovine serum (HyClone, GE Healthcare Life Science, USA) at 37 °C with 5% CO_2_ for 24 h. Subsequently, the culture medium was replaced with MEM containing 0.5% DMSO, along with PZQ, FAK inhibitor 14, and PF-03814735 at concentrations ranging from 10 ng/mL to 100 µg/mL, and then incubated for another 24 h. After incubation, 10 µL of a 5 mg/mL solution of 3-(4,5-dimethylthiazol-2-yl)-2,5-diphenyltetrazolium bromide (AppliChem GmbH, Germany) was added to each well and incubated for 4 h at 37 °C with 5% CO_2_. Following this, the solutions were replaced with a solvent mixture (4 mM HCl, 0.1% Nonidet P-40, in isopropanol) to dissolve the formazan crystals, and the absorbance was measured at 590 nm with a reference wavelength of 620 nm. The percentage of cell viability was determined by comparing the absorbance of the treated groups with that of the untreated control. The CC_50_ was calculated using GraphPad Prism 9 software. The cytotoxicity testing was done in triplicate experiments.

### Immunohistochemistry

Adult worms were fixed overnight at 4 °C in 10% neutral buffer formalin. The worms were dehydrated through an ethanol series and, therefore, infiltrated and embedded in graded paraffin. The embedded worms were cut into 4 μm thick section and placed on pre-coated immunohistochemistry slides. Heat-induced antigen retrieval with citrate buffer (pH 6) was used to enhance tissue antigenicity. The EnVision FLEX/HRP (K8002; DAKO, Denmark) and EnVision G/2 System/AP (K5355-11; DAKO, Denmark) kits were used for peroxidase and alkaline phosphatase staining systems, respectively. Subsequence to non-specific binding and endogenous peroxidase blocks, anti-phosphoserine (Merck, USA, AB1603) was applied to the tissue at 1:100 dilution. With regard to the staining systems, the tissue was then incubated in secondary antibody conjugation kits, visualized by either 3, 3-diaminobenzidine or liquid permanent red, and counter-stained by hematoxylin. Immunolocalization was examined under a light microscope (BX51, Olympus, Japan) with a digital camera (DP20, Olympus, Japan).

## Data availabilty

All data generated or analysed during this study are included in this published article (and its Supplementary Information files).

### Supplementary Information

Supplementary Information 1.

Supplementary Information 2.

Supplementary Information 3.
