# Supplementary material for: A reanalysis and integration of transcriptomics and proteomics datasets unveil novel drug targets for Mekong schistosomiasis
Source: Sci Rep. 2024 Jun 5;14:12969. doi: 10.1038/s41598-024-63869-0 (PMC11153569; doi:10.1038/s41598-024-63869-0)
Supplement: Supplementary file 3 — Supplementary Information 3. [file 41598_2024_63869_MOESM3_ESM.pdf]

Supplement

Multiple alignment with BLASTP algorithm

Tyrosine kinase (G4VP68) align against all database

Description	Accession	Max Score	Total Score	Query Cover	E value	% identity
Hypothetical protein CRM22_005195 [*Opisthorchis felineus*]	TGZ66668.1	538	538	73%	1.00E-178	56.28%
Protein-tyrosine kinase 2-beta [*Clonorchis sinensis*]	RJW69385.1	534	534	73%	0	56.84%
Hypothetical protein CRM22_005195 [*Opisthorchis felineus*]	TGZ66666.1	536	1073	73%	0	56.61%
PTK2 protein tyrosine kinase 2 [*Fasciolopsis buski*]	KAA0200331.1	554	554	73%	0	59.77%
Unnamed protein product [*Schistosoma margrebowiei*]	VDO52985.1	561	561	49%	0	90.72%
Focal adhesion kinase [*Fasciola hepatica*]	THD26862.1	573	573	73%	0	59.91%
Focal adhesion kinase 1 [*Schistosoma haematobium*]	XP_012798427.1	925	925	96%	0	78.60%
Tyrosine kinase [*Schistosoma mansoni*]	XP_018653840.1	939	939	96%	0	79.12%
Focal adhesion kinase 1 [*Schistosoma bovis*]	RTG87482.1	1011	1011	96%	0	82.65%
Focal adhesion kinase 1 [*Schistosoma japonicum*]	TNN08032.1	1098	1098	97%	0	92.68%

Tyrosine kinase (G4VP68) align against human database

Description	Accession	Max Score	Total Score	Query Cover	E value	% identity
Focal adhesion kinase 1 isoform X6 [*Homo sapiens*]	XP_024302971.1	323	323	80%	2.00E-97	37.43%
Focal adhesion kinase 1 isoform uu [*Homo sapiens*]	NP_001339672.1	320	320	80%	2.00E-97	37.11%
Focal adhesion kinase 1 isoform jj [*Homo sapiens*]	NP_001339671.1	320	320	80%	2.00E-97	37.11%
Focal adhesion kinase 1 isoform d [*Homo sapiens*]	NP_001303271.1	321	321	80%	1.00E-97	37.11%
PTK2 protein [*Homo sapiens*]	AAH35404.1	322	322	80%	1.00E-97	37.88%
Focal adhesion kinase 1 isoform p [*Homo sapiens*]	NP_001339646.1	323	323	80%	7.00E-98	37.43%
Focal adhesion kinase 1 isoform n [*Homo sapiens*]	NP_001339644.1	323	323	80%	7.00E-98	37.68%
Focal adhesion kinase 1 isoform ii [*Homo sapiens*]	NP_001339670.1	322	322	80%	4.00E-98	38.13%
Focal adhesion kinase 1 isoform dd [*Homo sapiens*]	NP_001339665.1	323	323	80%	2.00E-98	37.43%
Focal adhesion kinase 1 isoform hh [*Homo sapiens*]	NP_001339669.1	323	323	80%	1.00E-98	37.43%

Alpha subunit of casein kinase II (C1L7H2) align against all database

Description	Accession	Max Score	Total Score	Query Cover	E value	% identity
Alpha subunit of casein kinase II [*Fasciola gigantica*]	TPP63744.1	615	615	92%	0	84.49%
Casein kinase II subunit alpha [*Paragonimus westermani*]	KAA3679523.1	618	618	90%	0	89.40%
Alpha subunit of casein kinase II [*Fasciolopsis buski*]	KAA0196434.1	621	621	92%	0	86.06%
Hypothetical protein CRM22_006335 [*Opisthorchis felineus*]	TGZ64489.1	624	624	94%	0	86.81%
Hypothetical protein CRM22_006335 [*Opisthorchis felineus*]	TGZ64488.1	627	627	96%	0	85.68%
Unnamed protein product [*Schistosoma margrebowiei*]	VDO50886.1	627	627	90%	0	86.34%
Protein kinase [*Schistosoma mansoni*]	XP_018655235.1	733	733	92%	0	98.60%
Casein kinase II subunit alpha [*Schistosoma bovis*]	RTG88433.1	738	738	93%	0	98.33%
SJCHGC01351 protein [*Schistosoma japonicum*]	AAW27808.1	745	745	92%	0	99.44%
Casein kinase II subunit alpha [*Schistosoma japonicum*]	TNN07580.1	792	792	100%	0	98.44%

Alpha subunit of casein kinase II (C1L7H2) align against human database

Description	Accession	Max Score	Total Score	Query Cover	E value	% identity
Chain E, Casein Kinase Ii Subunit Alpha [*Homo sapiens*]	4MD7_E	540	540	92%	0	75.55
casein kinase II alpha subunit [*Homo sapiens*]	CAA49758.1	540	540	92%	0	75.27
unnamed protein product [*Homo sapiens*]	BAG35434.1	542	542	92%	0	75.27
casein kinase II alpha 1 subunit isoform a [*Homo sapiens*]	BAG70225.1	542	542	92%	0	75.27
CSNK2A1 protein [*Homo sapiens*]	AAH50036.1	543	543	92%	0	75.55
casein kinase II subunit alpha isoform a [*Homo sapiens*]	NP_001349699.1	543	543	92%	0	75.55
Chain A, Casein Kinase Ii Subunit Alpha [*Homo sapiens*]	5MOV_A	566	566	84%	0	80.56
Chain A, Casein kinase II subunit alpha [*Homo sapiens*]	5OSL_A	568	568	84%	0	80.37
Chain A, Casein Kinase Ii Subunit Alpha [*Homo sapiens*]	5CSP_A	568	568	84%	0	80.37
Chain A, Protein Kinase Ck2 [*Homo sapiens*]	1NA7_A	572	572	84%	0	80.98

Phosphoglycerate kinase (C1LT16) align against all database

Description	Accession	Max Score	Total Score	Query Cover	E value	% identity
Phosphoglycerate kinase [*Schistosoma japonicum*]	AAP74224.1	821	821	100%	0	96.19%
Phosphoglycerate kinase 1 [*Schistosoma japonicum*]	CAX71718.1	831	831	100%	0	97.84%
Phosphoglycerate kinase 1 [*Schistosoma japonicum*]	CAX71724.1	833	833	100%	0	97.84%
Phosphoglycerate kinase 1 [*Schistosoma japonicum*]	CAX77841.1	836	836	100%	0	98.08%
Phosphoglycerate kinase 1 [*Schistosoma japonicum*]	CAX77838.1	837	837	100%	0	98.08%
Phosphoglycerate kinase 1 [*Schistosoma japonicum*]	CAX71720.1	837	837	100%	0	98.08%
Phosphoglycerate kinase 1 [*Schistosoma japonicum*]	CAX71721.1	837	837	100%	0	98.08%
Phosphoglycerate kinase isoform 1 [*Schistosoma japonicum*]	TNN18745.1	837	837	100%	0	98.32%
Similar to phosphoglycerate kinase [*Schistosoma japonicum*]	AAP06480.1	838	838	100%	0	98.32%
Phosphoglycerate kinase 1 [*Schistosoma japonicum*]	CAX77844.1	838	838	100%	0	98.32%

Phosphoglycerate kinase (C1LT16) align against human database

Description	Accession	Max Score	Total Score	Query Cover	E value	% identity
Chain A, Phosphoglycerate Kinase K219a Mutant [*Homo sapiens*]	2X14_A	597	597	98%	0	70.87%
Chain A, Phosphoglycerate Kinase 1 [*Homo sapiens*]	4AXX_A	598	598	99%	0	70.91%
Chain A, Phosphoglycerate Kinase 1 [*Homo sapiens*]	2WZD_A	598	598	99%	0	70.91%
Chain A, Phosphoglycerate kinase 1 [*Homo sapiens*]	5M6Z_A	598	598	98%	0	70.87%
Chain A, Phosphoglycerate Kinase 1 [*Homo sapiens*]	4O33_A	598	598	99%	0	70.91%
Chain A, Phosphoglycerate kinase 1 [*Homo sapiens*]	5M3U_A	598	598	98%	0	70.87%
Chain A, PHOSPHOGLYCERATE KINASE 1 [*Homo sapiens*]	2Y3I_A	600	600	98%	0	71.12%
Chain A, Phosphoglycerate Kinase [*Homo sapiens*]	2ZGV_A	600	600	99%	0	71.15%
Chain A, Phosphoglycerate Kinase 1 [*Homo sapiens*]	2WZB_A	600	600	98%	0	71.12%
phosphoglycerate kinase 1 [*Homo sapiens*]	NP_000282.1	600	600	99%	0	71.15%
